# A Rare Case of Iatrogenic Diaphragm Defect following Laparoscopic Cholecystectomy Presented as Acute Respiratory Distress Syndrome

**DOI:** 10.1155/2018/4165842

**Published:** 2018-04-16

**Authors:** Konstantinos Bouchagier, Evangelos Solakis, Serafeim Klimopoulos, Theano Demesticha, Dimitrios Filippou, Panagiotis Skandalakis

**Affiliations:** ^1^2nd Surgical Department of General Hospital Evangelismos, Athens, Greece; ^2^Department of Anatomy, Medical School, University of Athens, Athens, Greece

## Abstract

Laparoscopic cholecystectomy is considered as the gold standard in the treatment of gallbladder disease. Laparoscopy presents significant advantages including decreased hospital stay, better aesthetic results, faster rehabilitation, less pain, reduced cost, and increased patient satisfaction. The complications' prevalence is low; however, the overall serious complication rate seems to be higher compared to open cholecystectomy, despite the increasing experience. Diaphragmatic injury following laparoscopic cholecystectomy is an extremely rare complication, and a high index of clinical suspicion is necessary to diagnose this situation that has a variety of clinical presentations and might be life-threatening. We present a unique case of postlaparoscopic cholecystectomy diaphragm defect with late onset. The clinical findings included those of respiratory distress syndrome along with small bowel incarceration and peritonitis.

## 1. Introduction

Laparoscopic cholecystectomy has been established as the gold standard in the elective management of cholelithiasis. Complications after laparoscopic cholecystectomy are reported in less than 5% of cases [1–5].Diaphragmatic injury is relatively uncommon, representing less than 1% of all traumatic injuries after laparoscopic cholecystectomy [6–8].

Penetrating mechanisms account for 65% of all diaphragmatic injuries, whereas blunt mechanisms account for the remaining 35% of them [7–9]. Overall morbidity associated with diaphragmatic injury ranges from 30 to 68% and is related to the presence of associated injuries. Moreover, blunt trauma diaphragmatic injuries have higher complication rates (herniation and pulmonary complications) (60%) in contrast to those with penetrating trauma (40%) [7–9].

We present a unique case of postlaparoscopic cholecystectomy diaphragm defect presenting as late-onset acute respiratory distress syndrome.

## 2. Case Report

**Figure 1:**
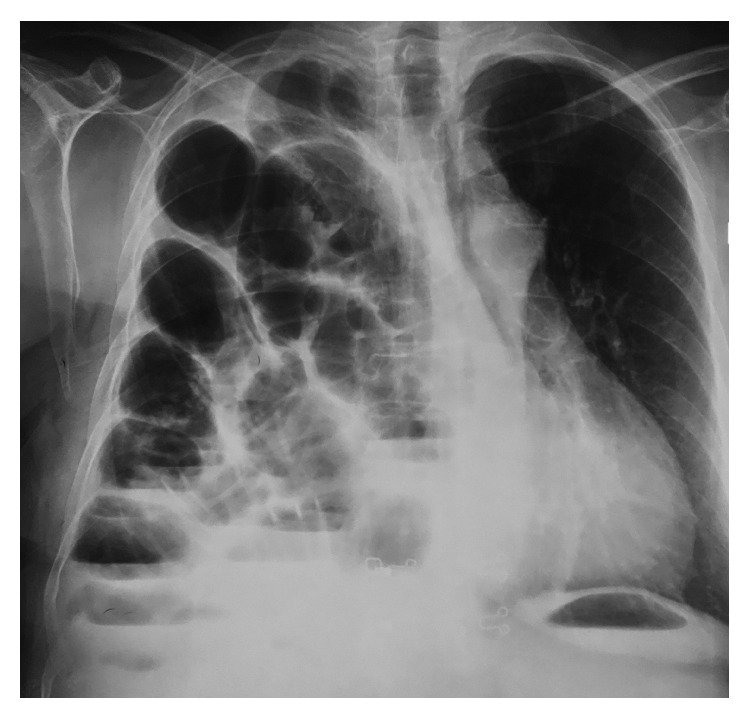
Chest X-ray performed immediately after admission to the Emergency Department. A large part of the small intestine has been transported to the right hemithorax causing functional right pneumonectomy. This image is in accordance with the clinical findings of the patient, which suggested acute respiratory distress syndrome.

**Figure 2:**
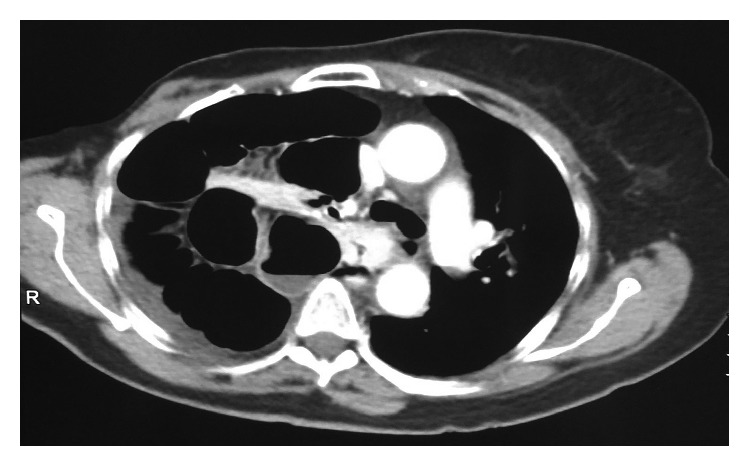
Abdomen and thorax CT scan. The findings confirmed the diagnosis of diaphragm traumatic herniation and the development of acute respiratory distress syndrome due to functional right pneumonectomy. Note that the right hemidiaphragm is absent and a large part of the small intestine is transported to the right side of the thoracic cavity.

A 58-year-old female was admitted to the emergency unit of our hospital suffering from acute dyspnoea, multiple episodes of vomiting, productive cough, and fever since 3 days. The patient reported a previous medical history of right mastectomy due to breast cancer followed by chemoradiotherapy and laparoscopic cholecystectomy for cholelithiasis ten months earlier. In the beginning of the operation after injection of anesthesia and endotracheal intubation, the patient developed pneumothorax which led to postponing the cholecystectomy. The pneumothorax was treated by inserting a thoracic drainage tube. After removing the thoracic drainage tube, chest X-ray and CT scan of the thorax were performed, but there was no evidence of diaphragm defect or herniation. Α laparoscopic cholecystectomy was performed four months later under general anesthesia with no complications. On admission to our department, the patient was febrile, pale, had considerable usage of accessory respiratory muscles, and had abdominal distention. On physical examination, the right lung presented decreased sounds during inspiration, while the abdomen was distended with diminished bowel sounds and peritonism. The blood exams were within normal values, while the arterial blood analysis demonstrated increased lactic acid levels and compromised pO_2_ values. Chest X-ray revealed that the right lung was totally suppressed by the intestine and suggested that due to intestinal transposition to the thoracic cavity, a functional right pneumonectomy had occurred (Figure 1). CT scan of the thorax and abdomen detected a right diaphragm defect and herniation of the small intestine, which was also ischaemic due to venous circulation occlusion (Figure 2). Urgent exploratory laparotomy was performed, and it revealed a diaphragm defect of 10 × 2 cm extending from the anterior side of the right diaphragm to the posterolateral side. Almost all the small intestine and part of the transverse colon were extending to the right thoracic cavity through the defect and were ischaemic and immobilized due to venous stasis. The herniated part of the small intestine and transverse colon were separated from the lung and retracted into the abdominal cavity, whereas the diaphragmatic defect was closed by direct suturing with Prolene 2-0 in two layers. Two vacuum drainage systems were placed in the right thoracic cavity and the right subphrenic area, subsequently. The abdominal wall was closed the next day after confirming the small and large intestine viability. The patient was hospitalized in the ICU for 11 days and was discharged from the hospital 20 days after the operation. The patient's postoperative course was complicated by an episode of acute myocardial ischaemia with increased troponin levels and mild ECG ischaemic changes which was treated successfully with beta blockers and dual antiplatelet agents. Mild fever due to retention of bronchial secretions and atelectasis was treated successfully by IV administered antibiotics. The patient was discharged the 20th postoperative day. A year after the operation, the patient remains free of symptoms and the postoperative CT of the thorax remains without findings.

## 3. Discussion

Diaphragmatic hernia is the herniation of intra-abdominal organs to the thoracic cavity, which may develop through a congenital defect or due to the diaphragm rupture after blunt or penetrating trauma. In this case, the iatrogenic diaphragm rupture may have been caused by laparoscopic instruments and xiphoid trocars, or after the thoracic tube insertion to treat pneumothorax in the first laparoscopy attempt that passed through the diaphragm and got widened over the period of time or maybe it was a diaphragmatic burst due to increased intra-abdominal pressure which is nearly impossible to occur, though. Most of diaphragmatic defects are asymptomatic or have a delayed clinical presentation, and high clinical suspicion needs to be maintained because herniation or strangulation of abdominal organs might be life-threatening [6–9]. The symptoms and signs depend on the size of the diaphragmatic defect, the part of the diaphragm that has a defect (right or left), the herniated organs, and the coexistent pulmonary disease. Symptomatic patients have abdominal pain, nausea, vomiting, chest pain, and dyspnoea, and in case of strangulation of the small or large intestine, abdominal distention with diminished intestinal sounds and peritonitis may occur [7–9]. In our case, the patient had a late onset of symptoms due to a diaphragm defect and presented symptoms of small bowel strangulation such as severe abdominal pain and abdominal distention with diminished bowel sounds. The interesting part of this case is the fact that, before the operation, we were not sure if it was a congenital or an acquired diaphragm rupture.

From an embryological point of view, congenital diaphragmatic hernias may be classified into four groups: diaphragmatic eventration, posterolateral hernia of Bochdalek, parasternal hernia of Morgagni-Larrey, and peritoneopericardial hernia [10]. Most diaphragmatic hernias are found on the left side because the pleuroperitoneal canal closes earlier, and moreover, the liver tamponades the right diaphragm. Congenital hernias usually present clinically in infants with respiratory distress syndrome that may need emergent laparotomy, whereas late onset on adults is rare and clinically discovered incidentally owing to gastrointestinal symptoms [10]. Thus, factors that favor the case of a congenital hernia are the site of the hernia on the parasternal anterior side and the late clinical onset of gastrointestinal symptoms.

The factors that favor the case of an iatrogenic hernia are the absence of a hernia sac which is expected on chronic hernias with adhesions, the negative radiologic images for diaphragm defects and herniation before the laparoscopic cholecystectomy, the late onset of clinical symptoms after a laparoscopic cholecystectomy, the severe nature of symptoms that led to urgent operation, and the considerable defect on the diaphragm extending from the anterior side to the posterolateral side. Furthermore, it must be taken into account that there could be a asymptomatic congenital hernia that became widened after intraperitoneal air insufflation, leading to intestinal herniation and respiratory distress syndrome, postoperatively [4, 5, 10].

Laparoscopic cholecystectomy is the optimal treatment for gallbladder pathology. The incidence of postoperative complications is 5% (in 2.6%, serious complications occur), and the most common complications are bile leak (“biloma”), bile duct injury, intra-abdominal abscess, wound infection, bleeding (liver surface and cystic artery are the most common sites), hernias, organ injury (intestine and liver are at highest risk, especially if the gallbladder has become adherent to other organs due to inflammation), deep vein thrombosis/pulmonary embolism, fatty acid, and fat-soluble vitamin malabsorption [2–4, 10].

Iatrogenic mechanical diaphragm ruptures with subsequent herniation of abdominal organs are extremely rare. In fact, searching worldwide literature, we found one case report by Armstrong et al. of a diaphragm hernia found six months after laparoscopic cholecystectomy, causing right-sided abdominal pain that was treated with right thoracotomy [1]. In our case, the symptoms' onset was ten months after the laparoscopic cholecystectomy contrary to the six months of the literature case. The clinical presentation of the presented case was with respiratory distress syndrome along with abdominal pain and peritonism due to incarceration and bowel ischaemia, in contrast to the literature case that the clinical presentation was with abdominal discomfort and pain. We proceeded to emergency laparotomy with abdominal incision, whereas Armstrong et al. proceeded to right thoracotomy. Moreover, we found a defect extending from the anterior side to the posterolateral side of the right hemidiaphragm, whereas Armstrong et al. found a small defect on the posterolateral side. The organ herniating through the defect was the whole small intestine in our case, contrary to fatty tissue found herniated and incarcerated by Armstrong et al. [1].

Taking into consideration the clinical presentation and because there is no relevant trauma history after the laparoscopic cholecystectomy that might have caused the diaphragm defect, we consider that this is a unique case of a postlaparoscopic cholecystectomy diaphragm defect that presented with respiratory consolidation.
